# Cerebral Amyloid Angiopathy—Related Inflammation: A Single-Center Experience and a Literature Review

**DOI:** 10.3390/jcm11226731

**Published:** 2022-11-14

**Authors:** Aikaterini Theodorou, Lina Palaiodimou, Apostolos Safouris, Odysseas Kargiotis, Klearchos Psychogios, Vasiliki Kotsali-Peteinelli, Aikaterini Foska, Vasiliki Zouvelou, Elias Tzavellas, Dimitrios Tzanetakos, Christina Zompola, John S. Tzartos, Konstantinos Voumvourakis, Georgios P. Paraskevas, Georgios Tsivgoulis

**Affiliations:** 1 Second Department of Neurology, “Attikon” University Hospital, School of Medicine, National and Kapodistrian University of Athens, 12462 Athens, Greece; 2 Stroke Unit, Metropolitan Hospital, Ethnarhou Makariou 9, N. Faliro, 18547 Piraeus, Greece; 3 First Department of Neurology, “Aiginition” Hospital, School of Medicine, National and Kapodistrian University of Athens, 11528 Athens, Greece; 4 First Department of Psychiatry, “Aiginition” Hospital, School of Medicine, National and Kapodistrian University of Athens, 11528 Athens, Greece; 5 Department of Neurology, University of Tennessee Health Science Center, Memphis, TN 38163, USA

**Keywords:** Cerebral Amyloid Angiopathy, inflammation, focal deficits, MRI, FLAIR, microbleeds, steroids, Apolipoprotein E

## Abstract

Background: Limited data exist regarding the prevalence of clinical, neuroimaging, and genetic markers among patients diagnosed with Cerebral Amyloid Angiopathy–related inflammation (CAA-ri). We sought to determine these characteristics in patients diagnosed in our center and to summarize available literature published either as single-case reports or small case series (<5 patients). Methods: We reported our single-center experience of patients diagnosed with CAA-ri according to international criteria during a seven-year period (2015–2022), and we abstracted data from 90 previously published cases. Results: Seven patients (43% women, mean age 70 ± 13 years) were diagnosed with CAA-ri in our center. The most common symptom at presentation was focal neurological dysfunction (71%), and the most prevalent radiological finding was the presence of T2/FLAIR white matter hyperintensities (100%). All patients were treated with corticosteroids and had a favorable functional outcome. Among 90 previously published CAA-ri cases (51% women, mean age 70 ± 9 years), focal neurological dysfunction was the most common symptom (76%), followed by a cognitive decline (46%) and headache (34%). The most prevalent neuroimaging findings were cerebral microbleeds (85%), asymmetric T2/FLAIR white matter hyperintensities (81%), and gadolinium-enhancing T1-lesions (37%). Genetic testing for the Apolipoprotein-E gene was available in 27 cases; 59% carried the APOE ε4/ε4 genotype. The majority of the published CAA-ri cases (78%) received corticosteroid monotherapy, while 17 patients (19%) were treated with additional immunosuppressive treatment. Favorable functional outcome following treatment was documented in 70% of patients. Conclusion: Improving the vigilance of clinicians regarding the early recognition and accurate diagnosis of CAA-ri is crucial for swift therapy initiation, which may result in improved functional outcomes.

## 1. Introduction 

Cerebral Amyloid Angiopathy-related inflammation (CAA-ri), a distinct subset of Cerebral Amyloid Angiopathy (CAA), represents a very rare clinical entity [1,2,3]. Histopathologically it results from vascular and perivascular nondestructive inflammatory infiltration related to the deposition of amyloid-beta (β-amyloid) within the walls of leptomeningeal and cortical blood vessels [4,5,6]. Two pathological subtypes of CAA-ri are now generally accepted: nondestructive perivascular inflammation (inflammatory CAA (ICAA)) and transmural or intramural inflammation (Aβ-related angiitis (ABRA)) [7].

The main presenting symptoms among patients with CAA-ri are typically headache, cognitive impairment, focal neurological signs, and even epileptic seizures and encephalopathy [8,9]. On neuroimaging, unifocal or multifocal, asymmetric T2/Fluid-attenuated inversion recovery (FLAIR) hyperintense white matter lesions are mainly revealed and are characteristically associated with numerous cortical or subcortical cerebral microbleeds [10]. Other radiological findings, including parenchymal or leptomeningeal gadolinium enhancement, cortical superficial siderosis, and lobar hemorrhage, are also frequently depicted [11]. The most common differential diagnoses that should be excluded are primary central nervous system angiitis or tumors (primary central nervous system lymphoma) [12,13]. 

Based on the clinical information and the increasing availability of Magnetic Resonance Imaging (MRI), in 2015, Auriel et al., proposed the revised criteria for the diagnosis of CAA-ri [14]. However, and mainly because of the rarity of the disorder, reaching this diagnosis remains a challenge for every clinician. This results in delayed treatment initiation and adversely influences the prognosis of CAA-ri patients. 

In view of the former considerations, we sought to determine the prevalence of clinical, radiological, and genetic findings in patients with CAA-ri from our tertiary care stroke center during a seven-year period and to summarize available literature published either as single-case reports or small case series (<5 patients). Our aim was to highlight the clinical burden of different clinical characteristics and neuroimaging markers among patients with CAA-ri and to assess their prevalence rates in order to enhance the clinical vigilance of clinicians regarding this rare entity. In contrast to previous efforts, including even large prospective cohort studies, in assessing the prevalence of CAA-ri characteristics, we have tried to collect and evaluate all provided data from the numerous case reports and small case series available in the literature.

## 2. Methods

### 2.1. Single-Center Case Series

This retrospective observational study was conducted at a tertiary care stroke center in Athens (Second Department of Neurology of the National & Kapodistrian University of Athens located at “ATTIKON” University Hospital) during a seven-year period (2015–2022). We retrospectively reviewed the medical and radiological records of all patients in our center who were diagnosed with CAA-ri, according to the Criteria proposed by Auriel et al. [14] during the study period. All patients with CAA-ri were followed longitudinally and were evaluated during routine visits in our dedicated outpatient stroke clinic. 

For all the patients, 3 Tesla MRI scans, which included T2/FLAIR, T1, Diffusion-Weighted Imaging (DWI), Susceptibility-Weighted Imaging (SWI), and T1-post-gadolinium sequences, were available for the initial evaluation and at follow-up one month after treatment initiation. Two independent neuroradiologists reviewed brain MRI scans. We abstracted data on demographic, clinical, radiological, and genetic characteristics, as well as treatments and outcomes of all included patients. A favorable functional outcome was defined as an improved modified Rankin Scale (MRS) score [15].

### 2.2. Literature Review

Our group has previously conducted a systematic review and meta-analysis of clinical, neuroimaging, and genetic markers of CAA-ri using data from prospective and retrospective cohort studies [16]. Single-case reports and case series with <5 patients were excluded from this meta-analysis. Thus, the aim of this systematic literature review was to identify all single-case reports and small (<5) case series that have previously been published. 

We searched MEDLINE and Scopus, using search strings that included the following terms: “CAA-ri” and “Cerebral Amyloid Angiopathy—related inflammation”. No language or other restrictions were applied. Our search spanned from the inception of each electronic database to 25 July 2022. We additionally searched reference lists of published articles manually, along with conference abstracts, to ensure the comprehensiveness of the bibliography. All retrieved studies were independently assessed by two reviewers (AT and LP), and any disagreements were resolved after discussion with a third tie-breaking evaluator (GT). We abstracted data on demographic, clinical, radiological, and genetic characteristics of all included case reports and case series.

Eligible studies were subjected to quality control and bias assessment employing the Joanna Briggs Institute Critical Appraisal Checklist for case reports [17].

Data extraction was performed in structured reports, including author names, date of publication, demographic, clinical, radiological, and genetic characteristics, as well as treatments and outcomes. 

### 2.3. Statistical Analysis

First, we assessed the pooled prevalence of characteristics, treatments, and outcomes of all patients included in the present single-center case series. Second, we also evaluated the pooled prevalence of the same variables among published CAA-ri case reports or small (<5) case series identified by the systematic literature search. 

Continuous variables were presented as mean with standard deviation (SD) in the case of the normal distribution that was assessed using the Kolmogorov–Smirnov Test. Continuous variables with skewed distribution were presented as median with interquartile ranges (IQR). Categorical variables were presented as the number of patients with the corresponding percentages and 95% Confidence Intervals (CI). The adjusted Wald method, which provides the best coverage for binomial CI when samples are less than ≈150, was used for the computation of 95% CI [18]. All statistical analyses were conducted using the R software version 1.4.1717 (R Foundation for Statistical Computing, Vienna, Austria) [19].

### 2.4. Ethical Approval and Patient Consent

This study was approved by the Ethics Committee of our Institution (Decision Number: EBΔ 499/8-9-2020). All patients provided written informed consent for the publication of this report in accordance with the Declaration of Helsinki in its currently applicable form. 

## 3. Results

### 3.1. Single-Center Case Series

A total of seven patients (50% women, mean age 70 ± 13 years) diagnosed with probable CAA-ri according to international criteria [14] were included in this study. Demographic, clinical, and neuroimaging characteristics, genetic markers, treatments, and outcomes are shown in Table 1. Only one of the diagnosed patients underwent a brain biopsy. Three patients gave their consent for genetic analysis of the Apolipoprotein E (APOE) gene, and one of them had the APOE ε4/ε4 genotype. The most common symptom at presentation was focal neurological dysfunction (71%), followed by mild headache (29%), encephalopathy (29%), seizures (14%), and cognitive decline (14%). One patient presented with visual hallucinations.

**Figure 1:**
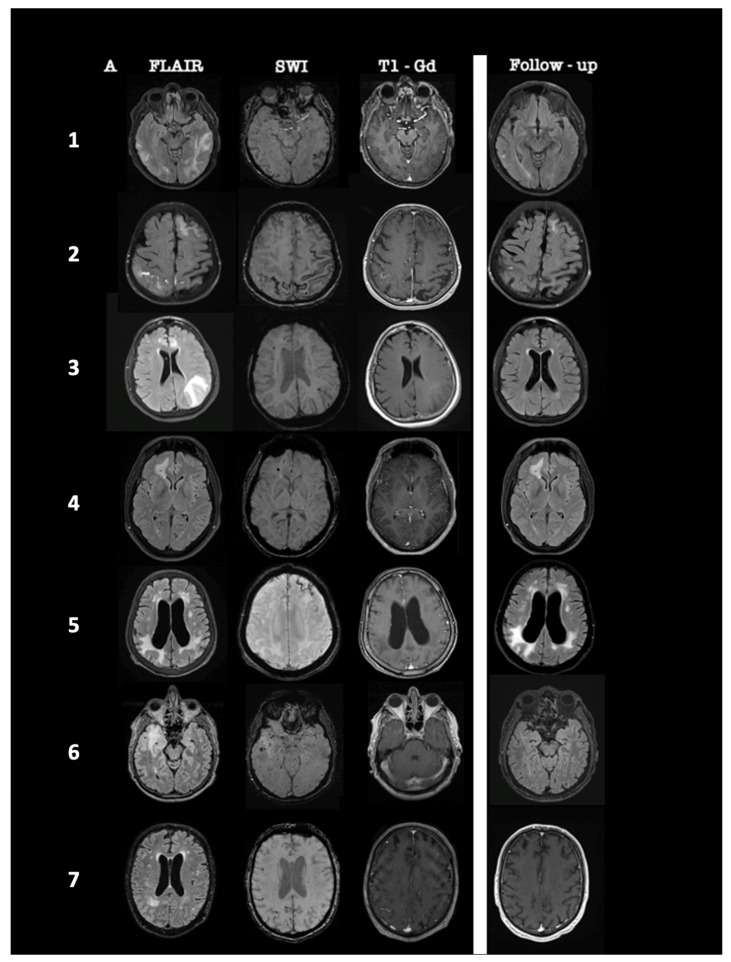
Summary of brain MRI characteristics of 7 patients at diagnosis and one month after treatment initiation. MRI findings of patients #1 to #7 are ordered from the top to the bottom. At diagnosis, axial fluid-attenuated inversion recovery (FLAIR) sequences showed supratentorial white matter lesions, multifocal in patients #1, #2, #3, and #5 and unifocal in patients #4, #6, and #7. In patients #1, #3, #4, #6, and #7, these lesions were associated with multiple cortical and subcortical cerebral microbleeds on Susceptibility Weighted Images, whereas patient #2 demonstrated left frontal cortical superficial siderosis (CSS) and subacute cortical subarachnoid hemorrhage (CSAH) on the right parietal lobe, and disseminated CSS was depicted on patient #5. White matter lesions on patients #2, #3, #4, #6, and #7 were associated with parenchymal or leptomeningeal contrast enhancement. In patients #1, #2, #3, #4, #5, and #6, follow-up axial FLAIR sequence one month after corticosteroid initiation showed a marked regression of hyperintense lesions on patients #1, #2, #3, and #6 and a stable size of hyperintense lesions on patients #4 and #5. In patient #7, the follow-up axial post-gadolinium T1 sequence revealed a complete resolution of gadolinium enhancement.

The most prevalent radiological findings were the following: T2/FLAIR white matter hyperintensities (100%, unifocal 43%, multifocal 57%), cerebral microbleeds (71%), and gadolinium-enhancing T1-lesions (71%). SWI sequences revealed disseminated cortical superficial siderosis (CSS) in one patient, whereas another patient presented with subacute convexity subarachnoid hemorrhage (CSAH) in the right parietal lobe. A summary of the MRI findings of all patients at diagnosis and 1-month follow-up post-treatment initiation is presented in Figure 1. 

All these patients were treated initially with a pulse corticosteroid therapy (1 g methylprednisolone intravenous once daily for five days), which resulted in a rapid clinical improvement in all of them. The pulse steroid course was followed in all cases by a slow steroid tapering regimen over several months under close clinical and imaging monitoring. 

Brain MRI was performed one month after treatment initiation, and in four patients #1, #2, #3, #6, the neuroimaging examination revealed a nearly complete resolution of the T2/FLAIR hyperintense white matter lesions. Two patients #4, #5 showed only partial or mild resolution of the white matter lesions, which remained stable in the next follow-up MRIs at 3, 6, and 12 months. In one patient #7, contrast-enhanced T1-weighted MRI sequences revealed complete resolution of gadolinium enhancement 3 months after corticosteroid initiation. Five patients #1, #2, #3, #4, #5 were clinically and radiologically stable after 12 and 24 months of follow-up, and one patient #2 died due to severe COVID-19 pulmonary infection 18 months after diagnosis. 

### 3.2. Literature Review

Seventy-one case reports [20,21,22,23,24,25,26,27,28,29,30,31,32,33,34,35,36,37,38,39,40,41,42,43,44,45,46,47,48,49,50,51,52,53,54,55,56,57,58,59,60,61,62,63,64,65,66,67,68,69,70,71,72,73,74,75,76,77,78,79,80,81,82,83,84,85,86,87,88,89,90] presenting 90 patients with CAA-ri (51% women, mean age 70 ± 9 years) were included in this systematic review (Supplementary Table S1). The risk of bias in the included case reports or small case series studies was assessed by the Joanna Briggs Institute Critical Appraisal Checklist for Case Reports and is presented in Supplementary Table S2 [17]. The overall score was 488 of 568 (86%), which is considered to be indicative of high quality. The excluded studies (case reports or small case series) with specific reasons for exclusion are listed in Supplementary Table S3 [91,92,93,94,95].

Demographic, clinical, and neuroimaging characteristics, genetic markers, treatments, and outcomes are shown in Table 2**.** Forty-six cases (51%; 95%CI: 41–61%) underwent a brain biopsy, and forty-two patients (47%; 95% CI: 37–57%) were diagnosed with definite CAA-ri, thirty-seven patients (41%; 95% CI: 32–52%) with probable, and eleven patients (12%; 95% CI: 7–21%) with possible CAA-ri according to the criteria of Auriel et al. [14]. APOE genotype was available in 27 patients, and 16 among these (59%; 95% CI: 41–76%) carried the APOE ε4/ε4 alleles.

Approximately three-fourths of the patients (*n* = 68; 76%; 95% CI: 66–83%) presented with focal neurological signs, and 46% had cognitive decline (*n* = 41; 95% CI: 36–56%) on presentation. One-third (*n* = 31; 34%; 95% CI: 25–45%) of patients complained about headaches on presentation, and the prevalence of encephalopathy, seizures, and psychiatric symptoms during baseline evaluation was 24% (*n* = 22; 95% CI: 17–34%), 24% (*n* = 22; 95% CI: 17–34%), and 22% (*n* = 20; 95% CI: 15–32%), respectively, in the included cases. Cortical and subcortical CMBs were the most common neuroimaging findings reported in 85% of included cases (*n* = 75; 95% CI: 74–90%). In seventy-three patients (81%; 95% CI: 72–88%), the pretreatment brain MRI revealed unifocal or multifocal asymmetric T2/FLAIR hyperintense white matter lesions, whereas parenchymal or leptomeningeal gadolinium-enhancement was documented in more than one-third (*n* = 33; 37%; 95% CI: 27–47%) of patients. CSS and acute lobar hemorrhage were detected in 17% (*n* = 15; 95% CI: 10–26%) and 13% (*n* = 12; 95% CI: 8–22%) of CAA-ri cases, respectively, and even more rarely cerebral infarctions were shown in only 9% of patients (*n* = 8; 95% CI: 4–17%).

More than 75% of the reported patients (*n* = 69; 95% CI: 67–84%) were treated with corticosteroid therapy after the diagnosis of CAA-ri, and about 20% of all patients (*n* = 17; 95% CI: 12–28%) received additionally immunosuppressive therapy because of the poor response to steroids. The additional immunosuppressive therapy was the following: cyclophosphamide (*n* = 9), mycophenolate mofetil (*n* = 5), methotrexate (*n* = 1), rituximab (*n* = 1), intravenous immunoglobulin (*n* = 1), or plasma exchange (*n* = 1). A favorable functional outcome with clinical and radiological improvement was reported in 70% of included cases (*n* = 63; 95% CI: 60–79%). 

## 4. Discussion

The early identification of CAA-ri is highly important, affecting the management, treatment, and outcome of these patients [96]. Diagnostic criteria for CAA-ri have been proposed, and the definite diagnosis is met if a histopathological examination is available, revealing a perivascular, transmural, or intramural, mostly nondestructive inflammation, in combination with β-amyloid deposition within the walls of cortical and leptomeningeal blood vessels [14]. A probable or possible CAA-ri diagnosis should be taken into consideration in patients 40 years of age or older with relevant acute or subacute clinical symptoms and characteristic neuroimaging findings. In these patients, other systemic diseases, including infections, brain tumors, autoimmune encephalopathies, or paraneoplastic syndromes, should be carefully excluded [13,97,98].

The present study, involving seven newly diagnosed and ninety previously reported cases with CAA-ri, demonstrates that the mean age of patients at diagnosis is about 70 years with a male-to-female-ratio of 1:1. Focal neurological signs were the most common clinical manifestations among our patients and the published case reports or case series. Cognitive decline was the second most frequent symptom in the previously published cases. The most prevalent neuroimaging findings included T2/FLAIR hyperintense white matter lesions, cerebral microbleeds, and gadolinium-enhancing T1-lesions. All our patients and 78% of the previously published cases received corticosteroid therapy, and approximately 70% had a favorable functional outcome. 

Our findings are in accordance with the existing literature. Indeed, with an average age of 67 years at diagnosis, CAA-ri is characterized as a disease of the elderly. Cognitive decline/dementia, focal neurological signs, headache, and seizures represent the most common clinical manifestations of the disease [5,99]. On MRI sequences, the most frequently depicted findings are the asymmetric white matter hyperintensities on T2/FLAIR sequences, the cortical or subcortical microbleeds on SWI, and the parenchymal or leptomeningeal gadolinium enhancement [9,100]. Interestingly, in CAA-ri patients, the distribution of neuroimaging findings, especially of cerebral microbleeds, does not follow the occipital dominance regional pattern of the CAA [3,61]. Additionally, a recent study demonstrated a higher amyloid burden of CAA-ri compared with other noninflammatory CAA, with a greater number of CMBs, resulting in a more severe disorder with worse clinical outcomes and a more significant loss of autonomy if CAA-ri remains untreated [96].

APOE ε4/ε4 homozygosity has been associated with a higher burden of β-amyloid deposition in cerebral vessel walls and a greater number of CMBs and therefore has been characterized as a risk factor for CAA-ri [101]. Previous studies have reported a high prevalence of ε4/ε4 (77%) among CAA-ri patients [102], whereas a recent large prospective cohort of patients with CAA-ri describes a much smaller (14%) prevalence of APOE ε4/ε4 homozygotes [8]. The pooled prevalence of the APOE ε4/ε4 genotype among the previously published cases was 59%, indicating that further prospective cohort studies are needed in order to investigate the genetic background of CAA-ri patients. 

In summary, an algorithm based on clinical suspicion would be crucial for the early diagnosis of CAA-ri [101]. The next step would take into account relevant neuroimaging findings, such as T2/FLAIR hyperintense white matter lesions complicated with cerebral microbleeds and gadolinium enhancement [103]. After excluding other possible differential diagnoses and probably collecting supporting findings such as the APOE ε4/ε4 homozygosity, the probable CAA-ri may be safely diagnosed according to International Criteria [14,104]. Notably, it should be highlighted that conventional open surgery or even the stereotactic biopsy has little value in the management of CAA-ri other than confirming the diagnosis in cases with diagnostic difficulties or impasse [37,105]. In the majority of the cases, the treatment initiation with corticosteroids should not be delayed, pending a biopsy [106,107].

Steroids have been primarily used for the treatment of patients with CAA-ri. In most of the published case reports or small case series, the patients showed rapid clinical and radiological responses, with low remission rates following corticosteroid pulse therapy [108,109,110]. This observation is in agreement with our single-center experience of rapid and persistent improvement in all our patients after a short intravenous course of steroids, followed by oral tapering. In the previous case reports, additional immunosuppressive therapies, including cyclophosphamide, plasma exchange, IVIG, or rituximab, have been used [20,22,28,30]. However, the optimal treatment duration of immunosuppressive therapy to avoid relapses remains to be defined. 

The present study has some limitations that should be acknowledged. First, the retrospective design and the small size of our cohort predispose us to selection biases. Second, only one of our patients and approximately 50% of the reviewed cases relied on histopathological confirmation of CAA-ri diagnosis. Third, a lack of consensus in diagnostic approach and therapeutic management among patients with CAA-ri could also confound our reported findings. Fourth, because of the methodological limitations associated with the pooling of data from case reports, further prospective validation of our results is required once larger cohorts and registries have been published. 

In conclusion, the present study documented that focal neurological signs and cognitive decline are the most common clinical features, and T2/FLAIR hyperintense white matter lesions, complicated with cerebral microbleeds and Gd+ enhancing lesions, were the most prevalent neuroimaging findings. To the best of our knowledge, this is the first effort to evaluate all the available individual case reports or small case series and to derive significant information regarding the main clinical and neuroimaging aspects of this entity. However, because of limited data from a small number of included patients, cautious interpretation of these results should be warranted. Despite the rarity of this disease, clinicians should be aware of the clinical manifestations and the neuroimaging characteristics of CAA-ri. Early diagnosis of CAA-ri may lead to swift therapy initiation that may impact favorably the overall prognosis of the patients. Finally, further prospective, multicenter cohort studies are needed to evaluate the prevalence rates of specific clinical and neuroimaging markers as well as genetic risk factors among patients with confirmed CAA-ri. 

## Figures and Tables

**Table 1:** Summary of the characteristics of patients diagnosed with Cerebral amyloid angiopathy-related inflammation at our center.

Sex/Age	Diagnosis *	Biopsy	History of ICH	APOE—Genotype	Clinical Features						MRI Findings						Treatment and Outcome			
					Psychiatric Symptoms	Encephalopathy	Focal Neurological Signs	Seizures	Cognitive Decline	Headache	Gd+ Enhancement	Unifocal FLAIR Lesions	Multifocal FLAIR Lesions	Microbleeds	Lobar Hemorrhage	CSS/CSAH	Corticosteroids	Other Immunosuppressive Therapy	Favorable Functional Outcome	Follow-Up Time
1 M/71	Pro	N	N	-	-	-	+	-	- 28/30	-	-	-	+	+	-	-	Yes	No	Yes	4 years
2 F/89	Pro	N	Y	-	+	+	-	-	-	-	+	-	+	-	-	SAH	Yes	No	Yes	18 months
3 F/73	Pro	N	N	ε4/ε4	-	-	+	-	-	+	+	-	+	+	-	-	Yes	No	Yes	3 years
4 M/45	Pro	N	N	ε3/ε3	-	-	+	-	- 30/30	-	+	+	-	+	-	-	Yes	No	Yes	2 years
5 F/77	Pro	N	Y	-	-	-	+	+	+	-	-	-	+	-	-	CSS	Yes	No	Yes	2 years
6 M/66	Pro	N	N	-	-	-	+	-	-	-	+	+	-	+	-	-	Yes	No	Yes	4 years
7 M/71	Def	Y	N	ε3/ε3	-	+	-	-	-	+	+	+	-	+	-	-	Yes	No	Yes	6 months

Abbreviations: F—Female, M—male, N—No, Y—Yes, Pro—Probable diagnosis, yrs—years, mo—months, CSS—Cortical Superficial Siderosis, CSAH—Convexity Subarachnoid Hemorrhage. * Diagnosis according to Auriel et al. criteria [14].

**Table 2:** Summary of the characteristics of the reported case reports and small case series with Cerebral Amyloid Angiopathy–related inflammation.

Variable	Previously Reported Cases	Present Case Series
No.	90	7
Age [years, mean (sd)]	70 (9)	70 (13)
Sex—Female, *n* (%, 95% CI)	46 (51%, 41–61%)	3 (43%)
Biopsy performed, *n* (%, 95% CI)	46 (51%, 41–61%)	1 (14%)
Definite/Probable/Possible CAA-ri *, *n* (%, 95% CI)	42 (47%, 37–57%)/ 37 (41%, 32–52%)/ 11 (12%, 7–21%)	
**Clinical Features, *n* (%)**		
Focal neurological signs, *n* (%, 95% CI)	68 (76%, 66–83%)	5 (71%)
Cognitive Decline, *n* (%, 95% CI)	41 (46%, 36–56%)	1 (14%)
Headache, *n* (%, 95% CI)	31 (34%, 25–45%)	2 (29%)
Encephalopathy, *n* (%, 95% CI)	22 (24%, 17–34%)	2 (29%)
Seizures, *n* (%, 95% CI)	22 (24%, 17–34%)	1 (14%)
Psychiatric Symptoms, *n* (%, 95% CI)	20 (22%, 15–32%)	1 (14%)
**MRI Findings, *n* (%)**		
T2/FLAIR Hyperintense White Matter Lesions (unifocal or multifocal), *n* (%, 95% CI)	73 (81%, 72–88%)	7 (100%)
Microbleeds, *n* (%, 95% CI)	75 (85%, 74–90%)	5 (71%)
Gd+ Enhancing Lesions, *n* (%, 95% CI)	33 (37%, 27–47%)	5 (71%)
cSS, *n* (%, 95% CI)	15 (17%, 10–26%)	1 (14%)
Lobar Hemorrhage, *n* (%, 95% CI)	12 (13%, 8–22%)	0 (0%)
Ischemic Infarcts, *n* (%, 95% CI)	8 (9%, 4–17%)	1 (14%)
**Treatment and Outcome, *n* (%)**		
Corticosteroids, *n* (%, 95% CI)	69 (78%, 67–84%)	7 (100%)
Corticosteroids plus **, *n* (%, 95% CI)	17 (19%, 12–28%)	0 (0%)
Favorable Outcome, *n* (%, 95% CI)	63 (70%, 60–79%)	7 (100%)
**APOE Genotype**		
APOE ε4/ε4—*n*/No (%)	16 / 27 (59%, 41–76%)	1/3 (33%)

* Diagnosis according to Auriel et al. criteria [14]. ** Additional therapies included cyclophosphamide, IVIG, plasma exchange, or rituximab.

## Data Availability

The datasets used and analysed during the current study are included in this article and its supplementary information files. More detailed datasets are available from the corresponding author on reasonable request.

## Supplementary Materials

The following supporting information can be downloaded at: https://www.mdpi.com/article/10.3390/jcm11226731/s1, Table S1: Table of case reports (*n* = 90) included in the systematic review; Table S2: Quality assessment of included case report studies (*n* = 71) according to the Joanna Briggs Institute Critical Appraisal Checklist for Case Reports; Table S3: Excluded Studies with Reasons for Exclusion.
