# Supplementary material for: Cerebral Amyloid Angiopathy—Related Inflammation: A Single-Center Experience and a Literature Review
Source: J Clin Med. 2022 Nov 14;11(22):6731. doi: 10.3390/jcm11226731 (PMC9692654; doi:10.3390/jcm11226731)
Supplement: Supplementary file 1 [file jcm-11-06731-s001.zip › jcm-2001893-supplementary.pdf]

TITLE: Cerebral Amyloid Angiopathy – related inflammation: a single-center experience and a literature review.

Supplementary Tables

Supplementary Table S1: Table of case reports (n=90) included in the systematic review.

Authors	Year	Diagnosis	Biopsy	Age	Sex	Prevalence at presentation						MRI Findings							Treatment		
						Psychiatric symptoms	Encephalopathy	Focal signs	Seizures	Cognitive decline	Headache	Infarcts	Gd enhancement	Unifocal	Multifocal	Microbleeds	ICH	cSS	Corticosteroids	Corticosteroids plus +	Favorable outcome
Aghetti, et al.[39]	2019	Def	Y	60	M	-	+	-	-	-	+	+	-	-	-	+	-	-	-	+	-
		Pro	Y	77	F	-	+	-	-	-	+	+	-	-	-	+	-	-	-	+	+
Alcalay, et al.[2]	2008	Pro	N	92	F	-	+	-	-	-	-	-	+	-	+	+	-	-	+	-	+
Alokley, et al.[40]	2021	Def	Y	67	F	+	+	+	+	-	+	-	+	-	+	+	+	+	-	+	+
Banerjee, et al.[4]	2018	Pro	N	62	M	-	-	-	-	-	+	-	-	-	+	+	-	-	+	-	+
		Pro	N	74	M	-	-	-	-	+	-	-	-	-	+	+	-	-	-	-	+
		Def	Y	54	F	-	-	+	-	-	+	-	+	+	-	+	-	-	+	-	+
Berkowitz, et al.[5]	2014	Pro	N	59	M	+	+	-	-	-	-	-	-	-	+	+	-	-	+	-	+
Boncoraglio, et al.[6]	2014	Def	Y	78	M	-	-	+	-	+	+	-	+	-	-	+	-	+	+	-	+
Caldas, et al.[7]	2015	Def	Y	63	M	+	-	+	-	+	+	-	-	-	+	+	-	-	-	+	-
		Def	Y	79	M	-	-	+	-	+	-	-	-	+	-	+	-	-	+	-	-
		Def	Y	70	M	-	-	+	+	-	-	-	-	+	-	+	-	-	+	-	-
Carmona-Iragui, et al.[8]	2015	Pos	N	73	M	-	-	+	-	+	-	-	-	-	+	+	-	+	+	-	-
		Pos	N	75	F	-	-	+	+	+	-	-	-	-	+	+	+	+	+	-	-
		Pos	N	68	F	-	-	+	-	+	-	-	-	-	+	+	-	-	+	-	-
		Pos	N	68	F	+	-	-	-	-	-	-	-	-	+	+	-	+	+	-	-
Cenina, et al.[41]	2015	Def	Y	65	F	-	-	+	-	+	-	-	+	-	+	+	-	+	-	+	+
Chen, et al.[10]	2022	Pro	N	73	M	-	+	+	-	-	-	+	+	-	+	+	-	-	-	+	+
Chung, et al.[42]	2010	Def	Y	83	F	-	+	-	+	+	-	-	-	-	+	NR	-	NR	+	-	-
		Def	Y	45	F	-	-	+	-	-	+	-	-	-	+	+	-	-	+	-	-

		Def	Y	72	M	-	-	+	+	-	-	-	-	-	+	NR	-	NR	-	+	-
**Crosta, et al.[12]**	**2015**	Pro	N	**NR**	M	-	+	+	+	+	-	-	-	-	+	+	-	-	+	-	-
**Daniels, et al.[13]**	**2009**	Pro	N	80	F	-	+	+	+	-	-	-	-	-	+	+	-	-	+	-	+
**DiFrancesco, et al.[14]**	**2011**	Pro	Y	68	M	+	-	-	-	+	-	-	-	-	+	+	-	-	+	-	+
**DiFrancesco, et al.[15]**	**2015**	Pro	N	74	F	-	-	+	-	-	-	-	+	-	+	+	-	-	+	-	+
		Pro	N	62	M	-	+	-	-	+	+	-	+	-	+	+	+	-	+	-	+
**Dörr, et al.[16]**	**2019**	Pro	N	71	M	+	-	+	-	+	-	-	+	-	+	+	+	+	+	-	-
**Du, et al.[17]**	**2019**	Pro	N	48	F	-	-	-	-	-	+	-	-	-	+	+	-	-	+	-	+
**Dudley, et al.[32]**	**2022**	Def	Y	77	F	+	+	+	-	-	+	-	+	-	+	+	-	-	+	-	+
**Ebiko, et al.[19]**	**2020**	Pos	N	68	F	-	-	+	-	-	-	-	+	-	+	+	+	-	+	-	-
**Esfahani-Bayerl, et al.[20]**	**2015**	Def	Y	70	M	+	-	+	-	+	-	-	-	-	+	+	-	-	+	-	+
**Fukasawa, et al.[21]**	**2015**	Def	Y	85	F	-	-	+	-	+	-	-	-	+	-	-	-	-	+	-	+
**Gera, et al.[22]**	**2019**	Pro	N	66	M	+	-	+	-	+	-	-	-	-	+	+	-	-	+	-	+
**Grasso, et al.[23]**	**2021**	Pro	N	71	F	+	-	+	-	-	-	-	+	-	+	+	-	-	-	+	-
		Pro	N	68	F	+	-	+	+	-	+	-	+	-	+	+	-	-	-	+	-
**Hagiwara, et al.[24]**	**2014**	Pro	Y	78	F	-	-	+	-	+	-	-	-	-	+	+	-	-	-	+	+
**Hainline, et al.[25]**	**2017**	Def	Y	73	F	-	-	+	-	-	+	-	+	+	-	-	+	-	+	-	+
**Holm-Yildiz, et al.[26]**	**2014**	Pro	N	79	M	-	-	+	-	-	+	-	-	-	+	+	-	-	+	-	-
**Iwanaga, et al.[27]**	**2012**	Def	Y	86	F	-	-	+	+	+	-	-	-	-	+	+	-	-	+	-	+
**Jeanneret, et al.[28]**	**2022**	Def	Y	55	F	-	-	+	-	-	+	-	+	-	+	+	-	-	+	-	+
**Kang, et al.[29]**	**2017**	Def	Y	69	F	-	-	-	-	+	-	-	+	-	+	-	-	-	NR	NR	NR
**Kimura, et al.[30]**	**2013**	Def	Y	72	F	-	+	+	-	-	+	-	-	-	+	-	-	-	-	+	+

**Kloppenborg, et al.[31]**	**2010**	Def	Y	74	M	-	+	+	+	+	-	-	+	-	+	+	-	-	+	-	+
**Kusakabe, et al.[32]**	**2018**	Def	Y	78	M	+	-	+	+	-	-	-	-	+	-	+	-	-	+	-	+
**Kuwahara, et al.[33]**	**2022**	Def	Y	80	F	-	-	+	-	+	-	-	-	-	+	+	-	-	+	-	+
**Liang, et al.[34]**	**2015**	Def	Y	76	F	-	-	+	-	-	+	-	+	-	-	-	-	-	+	-	+
**Machida, et al.[35]**	**2008**	Def	Y	69	F	-	+	+	-	-	-	-	+	-	+	-	-	+	+	-	+
**Makarewicz, et al.[36]**	**2019**	Def	Y	58	M	-	-	+	+	-	-	-	-	-	+	+	-	-	-	+	-
**Malhotra, et al.[37]**	**2016**	Def	N	57	M	-	-	-	-	-	+	-	+	-	+	-	-	+	+	-	+
**Maramattom, et al.[38]**	**2022**	Pos	N	77	F	-	+	+	-	-	-	-	+	-	+	+	-	-	+	-	+
**Maruyama, et al.[39]**	**2022**	Def	Y	65	F	-	-	+	+	-	+	-	-	-	+	-	+	+	-	-	-
**Masrori, et al.[40]**	**2019**	Def	Y	71	F	+	-	-	-	+	+	-	-	-	+	+	-	-	+	-	+
**Mendonca, et al.[41]**	**2015**	Pro	N	75	F	-	-	+	-	-	-	-	-	-	+	+	-	-	+	-	+
**Nada, et al.[26]**	**2021**	Def	Y	71	M	-	-	+	-	+	+	-	+	-	+	+	-	-	+	-	+
		Pro	N	73	F	-	+	+	-	-	-	-	-	-	+	+	-	-	+	-	+
		Pro	N	65	M	-	-	+	+	-	-	-	-	-	+	+	-	-	+	-	+
**Nelson, et al.[43]**	**2019**	Def	Y	57	M	+	-	+	+	-	+	-	-	-	+	+	-	-	+	-	+
**Onomura, et al.[44]**	**2021**	Pos	N	60	M	-	-	+	-	-	-	-	+	-	+	+	-	-	+	-	+
**Poli, et al.[45]**	**2019**	Pro	N	79	M	+	-	+	-	+	-	-	+	-	+	+	+	+	-	+	-
		Pro	N	76	F	+	-	+	-	+	+	-	+	-	+	+	-	+	+	-	+
**Rajczewska, et al.[46]**	**2018**	Pro	N	86	F	-	+	+	-	-	-	-	-	-	+	+	-	-	+	-	+
**Rastogi, et al.[47]**	**2015**	Pos	N	68	F	-	+	+	-	+	-	+	-	-	+	+	+	-	+	-	+
**Renard, et al.[48]**	**2017**	Pro	N	72	M	-	-	+	-	-	-	+	-	+	-	+	-	-	+	-	+
		Pro	N	86	F	-	-	+	-	-	-	-	-	-	+	+	+	-	-	+	-
		Pro	N	84	M	-	-	+	-	-	-	-	-	-	+	+	+	-	-	+	+

**Ribeiro, et al.[49]**	**2019**	Def	Y	83	F	-	-	+	-	-	+	-	-	-	+	+	-	-	+	-	+
**Rigney , et al.[50]**	**2015**	Pro	N	65	F	-	+	+	-	-	-	-	+	-	+	-	-	-	+	-	+
**Ringman, et al.[51]**	**2021**	Def	Y	64	M	-	-	-	-	+	-	-	-	+	-	-	-	-	+	-	-
**Roca, et al.[52]**	**2021**	Def	Y	54	M	-	+	+	+	-	-	-	-	+	-	+	-	-	+	-	+
**Sakaguchi, et al.[53]**	**2011**	Def	Y	56	M	-	-	+	+	+	-	-	+	+	-	-	-	-	+	-	+
**Sakai, et al.[54]**	**2011**	Def	Y	71	M	-	-	+	-	-	+	-	+	-	+	-	-	-	+	-	-
**Salam, et al.[55]**	**2017**	Def	Y	63	M	+	-	+	-	+	+	-	+	-	+	+	-	-	-	+	-
**Saliou, et al.[56]**	**2018**	Def	Y	61	M	-	-	+	-	+	-	+	-	-	+	+	-	-	+	-	+
**Salleles, et al.[57]**	**2019**	Def	Y	65	M	+	-	-	-	+	+	+	+	-	+	+	+	+	-	+	+
**Savoiardo, et al.[58]**	**2010**	Pro	N	76	M	-	-	-	-	+	-	-	-	-	+	+	-	-	+	-	+
**Savoiardo, et al.[59]**	**2011**	Pro	N	76	M	-	-	-	-	+	-	-	-	-	+	+	-	-	+	-	+
		Pro	Y	67	M	+	-	-	-	+	-	-	-	-	+	+	-	-	+	-	+
		Pro	N	68	F	-	-	+	-	-	-	-	-	+	-	+	-	-	+	-	+
**Severijns, et al.[60]**	**2022**	Def	Y	62	M	-	+	+	+	-	+	-	-	-	+	+	-	-	+	-	+
**Silek, et al.[61]**	**2018**	Pro	N	82	M	-	-	+	-	+	+	-	-	-	+	+	-	+	+	-	+
**Sowanou, et al.[62]**	**2022**	Pro	N	81	M	-	+	+	+	-	+	+	+	-	+	+	-	+	+	-	+
**Takeuchi, et al.[63]**	**2021**	Pos	Y	76	F	-	-	-	+	+	-	-	+	-	+	+	-	-	+	-	-
**Tetsuka, et al.[64]**	**2019**	Pro	N	77	F	-	-	+	-	-	-	-	-	-	+	+	-	-	-	-	+
**Tolchin, et al.[65]**	**2016**	Def	Y	52	M	+	-	+	+	-	-	-	+	-	+	+	-	-	+	-	+
**Tominaga, et al.[66]**	**2020**	Def	Y	57	F	-	-	-	+	+	-	-	-	-	+	+	-	-	+	-	-
**Voicu, et al.[67]**	**2021**	Pro	N	70	M	-	-	+	-	+	+	-	-	+	-	+	-	-	+	-	+
**Wengert, et al.[68]**	**2012**	Pos	N	83	F	-	-	+	-	+	-	-	-	-	+	+	-	-	+	-	+

**Xu, et al.[69]**	**2019**	Pro	N	69	F	-	-	-	-	+	-	-	-	-	+	+	-	-	+	-	+
**Yamamoto, et al.[70]**	**2020**	Def	Y	73	F	-	-	+	-	-	-	-	-	-	+	-	-	-	+	-	+
**Yamashita, et al.[71]**	**2020**	Pos	N	66	F	-	-	+	-	+	-	-	-	+	-	+	-	-	+	-	+

Abbreviations: Def: Definite Diagnosis, F: Female, M: Male, N: no, NR: not Reported, Pos: Possible Diagnosis, Pro: Probable Diagnosis, Y: yes

Supplementary Table S2: Quality assessment of included case report studies (n=71) according to the Joanna Briggs Institute Critical Appraisal Checklist for Case Reports.[17]

Study	Year	Demographics Characteristics	Patient History	Current Clinical Condition	Assessment	Intervention	Post-Intervention	Adverse Events	Takeaway Lessons	Overall Score †
**Aghetti, et al.[39]**	**2019**	1	1	1	1	1	1	0	1	7
**Alcalay, et al.[2]**	**2008**	1	1	1	1	1	1	0	1	7
**Alokley, et al.[40]**	**2021**	1	1	1	1	1	1	0	1	7
**Banerjee, et al.[4]**	**2018**	1	0	1	1	1	1	0	1	6
**Berkowitz, et al.[5]**	**2014**	1	0	1	1	1	1	0	1	6
**Boncoraglio, et al.[6]**	**2014**	1	1	1	1	1	1	0	1	7
**Caldas, et al.[7]**	**2015**	1	1	1	1	1	1	0	1	7
**Carmona-Iragui, et al.[8]**	**2015**	1	1	1	1	1	1	1	1	8
**Cenina, et al.[41]**	**2015**	1	0	1	1	1	1	0	1	6
**Chen, et al.[10]**	**2022**	1	1	1	1	1	1	0	1	7
**Chung, et al.[42]**	**2010**	1	1	1	1	1	1	0	1	7
**Crosta, et al.[12]**	**2015**	0	1	1	1	1	1	1	1	7
**Daniels, et al.[13]**	**2009**	1	0	1	1	1	1	0	1	6
**DiFrancesco, et al.[14]**	**2011**	1	1	1	1	1	1	0	1	7
**DiFrancesco, et al.[15]**	**2015**	1	1	1	1	1	1	0	1	7
**Dörr, et al.[16]**	**2019**	1	1	1	1	1	1	1	1	8
**Du, et al.[17]**	**2019**	1	1	1	1	1	1	0	1	7
**Dudley, et al.[18]**	**2022**	1	1	1	1	1	1	0	1	7
**Ebiko, et al.[19]**	**2020**	1	1	1	1	1	1	0	1	7

**Esfahani-Bayerl, et al.**[20]	**2015**	1	1	1	1	1	1	0	1	7
**Fukasawa, et al.**[21]	**2015**	1	0	1	1	1	1	0	1	6
**Gera, et al.**[22]	**2019**	1	0	1	1	1	1	0	1	6
**Grasso, et al.**[23]	**2021**	1	0	1	1	1	1	1	1	7
**Hagiwara, et al.**[24]	**2014**	1	0	1	1	1	1	0	1	6
**Hainline, et al.**[25]	**2017**	1	1	1	1	1	1	0	1	7
**Holm-Yildiz, et al.**[26]	**2014**	1	0	1	1	1	1	0	1	6
**Iwanaga, et al.**[27]	**2012**	1	0	1	1	1	1	0	1	6
**Jeanneret, et al.**[28]	**2022**	1	1	1	1	1	1	0	1	7
**Kang, et al.**[29]	**2017**	1	0	1	1	0	0	0	1	4
**Kimura, et al.**[30]	**2013**	1	1	1	1	1	1	0	1	7
**Kloppenborg, et al.**[31]	**2010**	1	1	1	1	1	1	0	1	7
**Kusakabe, et al.**[32]	**2018**	1	1	1	1	1	1	0	1	7
**Kuwahara, et al.**[33]	**2022**	1	0	1	1	1	1	1	1	7
**Liang, et al.**[34]	**2015**	1	1	1	1	1	1	1	1	8
**Machida, et al.**[35]	**2008**	1	1	1	1	1	1	1	1	8
**Makarewicz, et al.**[36]	**2019**	1	1	1	1	1	1	1	1	8
**Malhotra, et al.**[37]	**2016**	1	1	1	1	1	1	1	1	8
**Maramattom, et al.**[38]	**2022**	1	0	1	1	0	0	0	1	4
**Maruyama, et al.**[39]	**2022**	1	1	1	1	1	1	1	1	8
**Masrori, et al.**[40]	**2019**	1	1	1	1	1	1	1	1	8
**Mendonca, et al.**[41]	**2015**	1	1	1	1	1	1	0	1	7
**Nada, et al.**[26]	**2021**	1	1	1	1	1	1	0	1	7
**Nelson, et al.**[43]	**2019**	1	1	1	1	1	1	0	1	7
**Onomura, et al.**[44]	**2021**	1	1	1	1	1	1	0	1	7
**Poli, et al.**[45]	**2019**	1	1	1	1	1	1	0	1	7
**Rajczewska, et al.**[46]	**2018**	1	1	1	1	1	1	1	1	8
**Rastogi, et al.**[47]	**2015**	1	1	1	1	1	1	0	1	7
**Renard, et al.**[48]	**2017**	1	0	1	1	1	1	1	1	7
**Ribeiro, et al.**[49]	**2019**	1	1	1	1	1	1	0	1	7
**Rigney , et al.**[50]	**2015**	1	1	1	1	1	1	0	1	7
**Ringman, et al.**[51]	**2021**	1	1	1	1	1	1	1	1	8
**Roca, et al.**[52]	**2021**	1	1	1	1	1	1	0	1	7
**Sakaguchi, et al.**[53]	**2011**	1	1	1	1	1	1	0	1	7
**Sakai, et al.**[54]	**2011**	1	1	1	1	1	1	0	1	7
**Salam, et al.**[55]	**2017**	1	1	1	1	1	1	1	1	8
**Saliou, et al.**[56]	**2018**	1	1	1	1	1	1	0	1	7
**Salleles, et al.**[57]	**2019**	1	1	1	1	1	1	0	1	7
**Savoiardo, et al.**[58]	**2010**	1	1	1	1	1	1	0	1	7
**Savoiardo, et al.**[59]	**2011**	1	0	1	1	1	1	0	1	6
**Severijns, et al.**[60]	**2022**	1	1	1	1	1	1	0	1	7
**Silek, et al.**[61]	**2018**	1	1	1	1	1	1	1	1	8
**Sowanou, et al.**[62]	**2022**	1	1	1	1	1	1	0	1	7
**Takeuchi, et al.**[63]	**2021**	1	1	1	1	1	1	0	1	7

**Tetsuka, et al.[64]**	**2019**	1	1	1	1	1	0	0	1	6
**Tolchin, et al.[65]**	**2016**	1	1	1	1	1	1	0	1	7
**Tominaga, et al.[66]**	**2020**	1	1	1	1	1	1	0	1	7
**Voicu, et al.[67]**	**2021**	1	0	1	1	1	1	0	1	6
**Wengert, et al.[68]**	**2012**	1	0	1	1	1	1	0	1	6
**Xu, et al.[69]**	**2019**	1	1	1	1	1	1	0	1	7
**Yamamoto, et al.[70]**	**2020**	1	0	1	1	1	1	0	1	6
**Yamashita, et al.[71]**	**2020**	1	0	1	1	1	1	0	1	6
**Overall score**		70/71	52/71	71/71	71/71	69/71	68/71	16/71	71/71	488/568

†All studies presented thoroughly the patients' current clinical condition, the complete assessment for their cases and discussed takeaway lessons. However, one study did not present the patient's demographics[12] and nineteen studies did not present the previous patient history and concomitant diseases.[4,5,9,13, 21-24, 26,27,29, 33, 38, 48, 58, 67, 68, 70, 71] Additionally, two studies did not provide the diagnostic or therapeutic interventions used in their cases. [29,38] The post-intervention course was not discussed by three studies [29,38, 64] and the adverse events were discussed only in sixteen studies [8, 12, 16, 23, 33-37, 39, 40, 46, 48, 51, 55, 61]

<u>Supplemental Table S3:</u> Excluded Studies with Reasons for Exclusion

**Author**	**Year**	**Reason for exclusion**
Ng, et al.[73]	2017	Reported ABRA angiitis, not CAA-ri
Theodorou, et al.[74]	2019	Case included in the cases of the center
Koudriavtseva, et al.[75]	2021	CAA-ri, probable as adverse event of a novel therapy
Ray, et al.[76]	2022	CAA-ri, probable as adverse event of a novel therapy
Maddox, et al.[77]	2022	Full article was not found
